# Dosimetric evaluation of magnetic resonance-generated synthetic CT for radiation treatment of rectal cancer

**DOI:** 10.1371/journal.pone.0190883

**Published:** 2018-01-05

**Authors:** Hesheng Wang, Kevin Du, Juliet Qu, Hersh Chandarana, Indra J. Das

**Affiliations:** 1 Department of Radiation Oncology, New York University School of Medicine, New York, NY, United States of America; 2 Bernard and Irene Schwartz Center for Biomedical Imaging, Department of Radiology, New York University School of Medicine, New York, NY, United States of America; North Shore Long Island Jewish Health System, UNITED STATES

## Abstract

**Purpose:**

The purpose of this study was to assess the dosimetric equivalence of magnetic resonance (MR)-generated synthetic CT (synCT) and simulation CT for treatment planning in radiotherapy of rectal cancer.

**Methods:**

This study was conducted on eleven patients who underwent whole-body PET/MR and PET/CT examination in a prospective IRB-approved study. For each patient synCT was generated from Dixon MR using a model-based method. Standard treatment planning directives were used to create a four-field box (4F), an oblique four-field (O4F) and a volumetric modulated arc therapy (VMAT) plan on synCT for treatment of rectal cancer. The plans were recalculated on CT with the same monitor units (MUs) as that of synCT. Dose-volume metrics of planning target volume (PTV) and organs at risk (OARs) as well as gamma analysis of dose distributions were evaluated to quantify the difference between synCT and CT plans. All plans were calculated using the analytical anisotropic algorithm (AAA). The VMAT plans on synCT and CT were also calculated using the Acuros XB algorithm for comparison with the AAA calculation.

**Results:**

Medians of absolute differences in PTV metrics between synCT and CT plans were 0.2%, 0.2% and 0.3% for 4F, O4F and VMAT respectively. No significant differences were observed in OAR dose metrics including bladder V40Gy, mean dose in bladder, bowel V45Gy and femoral head V30Gy in any techniques. Gamma analysis with 2%/2mm dose difference/distance to agreement criteria showed median passing rates of 99.8% (range: 98.5 to 100%), 99.9% (97.2 to 100%), and 99.9% (99.4 to 100%) for 4F, O4F and VMAT, respectively. Using Acuros XB dose calculation, 2%/2mm gamma analysis generated a passing rate of 99.2% (97.7 to 99.9%) for VMAT plans.

**Conclusion:**

SynCT enabled dose calculation equivalent to conventional CT for treatment planning of 3D conformal treatment as well as VMAT of rectal cancer. The dosimetric agreement between synCT and CT calculated doses demonstrated the potential of MR-only treatment planning for rectal cancer using MR generated synCT.

## Introduction

Colorectal cancer is the third most common cancer in American men and women. It is estimated that 39,910 new cases of rectal cancer will be diagnosed in United States in 2017 [1]. While colorectal cancer incidence in USA is declining overall, the cancer is increasing rapidly among young adults, and nearly one-third of rectal cancer patients are younger than age 55 [2]. Radiation therapy (RT) is an important adjunct in the treatment of rectal cancer [3, 4]. Preoperative chemoradiotherapy is considered as the standard of care for patients with stage II and III rectal cancer. Short-course preoperative radiotherapy appears to be effective in tumor control and reduce the risk of local recurrence [5].

Magnetic resonance imaging is the most accurate tool for the local staging of rectal cancer due to superior soft tissue visualization [6, 7]. Meanwhile, MR has shown great prognostic power for treatment outcome in rectal cancer [7–9], and become a powerful tool in selection of appropriate treatment [10]. Although MR is the modality of choice for rectal cancer, it has been rarely used solely in its radiotherapy workflow. Conventional radiotherapy employs computed tomography (CT) for treatment planning due to the geometrical accuracy and direct correlation with tissue electron density of CT images. To utilize MR for disease assessment and tumor delineation in RT, MR images are first registered to CT simulation images, which is susceptible to systematic uncertainties [11]. Recently, the MR-only radiotherapy is gaining momentum with emerging MR-LINAC technology [12–14]. The MR-only treatment planning can avoid potential error in MR-to-CT registration, and spare the cost and radiation from dedicated CT simulation. The emerging hybrid MR and linear accelerator technique [15] also motivate MR-only treatment planning to simplify the process of MR guidance in RT.

A CT image directly provides a map of electron density that is necessary for accurate dose calculation [16]. However, there is no simple conversion from MR intensity to electron density value. To address the issue, techniques that generate synthetic CT (synCT) images from MR data are being developed [14, 17], and have shown promising results in treatment planning including brain [18], liver [19], prostate [20], and pelvic tumors [21]. These methods typically can be classified as atlas-based and classification-based approaches. Atlas-based methods search a template MR from an atlas of paired MR and CT data that optimally aligns with patient MR images, and then use the corresponding atlas CT for dose calculation [17]. These methods still rely on image registration, and ignore potential differences in anatomy and electron densities between patients. Classification-based methods use segmentation approach to MR images into several tissue classes and assign bulky densities to form a pseudo-CT image [22, 23]. However, these methods have difficulty in differentiating between bone and air as both air and bone show low signal in MR images.

A hybrid method that combines image classification and atlas delineation [24] recently has been evaluated to generate synCT from MR for treatment planning in lung cancer [25]. In this study, we investigated the potential of the hybrid method to create synCT for MR-based treatment planning of rectal cancer for both 3D conformal RT (3DCRT) and volumetric modulated arc therapy (VMAT). We hypothesized that the MR-generated synCT could provide equivalent dose calculation as simulation CT in colon and rectum cancer where substantial tissue inhomogeneities are present.

## Methods and materials

### Patient imaging

Under an Institutional Review Board (IRB)-approved protocol, PET/CT and subsequent whole-body PET/MR examination were performed for a clinical imaging study. The data for this work were retrospectively obtained from eleven patients (9 female, 2 male, age range: 25–75 years old) in the study. All image data was de-identified by anonymization and analyzed retrospectively.

The CT data were acquired on a PET/CT scanner (Biograph mCT; Siemens AG Healthcare) according to the standard clinical protocol. The free-breathing CT was acquired using a source of 100-140kVp for a volume with a pixel size of 1.52 × 1.52 mm^2^ or 1.37 × 1.37 mm^2^ and a slice thickness of 5.0 mm. The patients were subsequently scanned on a 3T whole-body PET/MR system (Biograph mMR; Siemens Healthcare) that integrates a PET detector with z-direction field of view of 25.8 cm in MR coils for hybrid imaging. In this study, the Dixon 2-point, 3-dimensional volume-interpolated (VIBE) sequence images [26] (TR/TE/Flip Angle: 3.6ms/2.46ms/10°) were acquired during patient breath-hold. The acquisition lasted for 19 sec and generated a 128-slice volume with 192 × 126 pixels in the coronal direction (resolution: 2.60 × 2.60 mm, slice thickness: 3.12mm).

### Synthetic CT generation

The synCT was generated from the Dixon MR images using a model-based method that was developed to create linear attenuation coefficients (LACs) map from MR for PET attenuation correction [24]. Detail of this method can be found elsewhere [24]. Briefly, the Dixon MR images were first segmented into four tissue classes of air, fat, lung and soft tissue using a soft-tissue segmentation algorithm [27]. Subsequently, the method [24] updated bone structure by using an atlas-based method that registered patient MR images to an atlas. The atlas consisted of a set of aligned MR image and bone mask pairs. Instead of binary bone type, the bone masks were voxels of actual bone intensities. The bone mask paired with atlas MR that optimally matched with patient MR was transferred back to patient image space based on the registration, thereby, added bone into the tissue class map to form final LAC map.

The LAC map was at the PET energy level of 511 keV. To create synCT image from a standard CT simulator at 140 kVp, voxel-by-voxel mapping of the LAC image to a synCT was performed by using a linear conversion [28]: LAC = 0.096 × (1 × 10^−3^ × HU + 1) for HU ≤ 0; otherwise LAC = 0.096 × (1 + 6.40 × 10^−4^ × HU), where LAC is in unit of cm^-1^. The tissue type air, fat, and soft tissue had HU numbers of -1000, -110 and 70, respectively; and the bone that was transferred from a bone mask had HU in the range of 70 to 2661 [25].

### Data preparation

To compare synCT and CT for treatment planning, synCT and CT images were expected to be perfectly aligned. We first performed an affine registration between the whole-body synCT and CT, and formatted both images into axial direction that is default setting for simulation CT. Subsequently, we cropped both volumes to the anatomy range typically requested by physicians in simulation for rectal cancer that covered from the interspace between vertebrate L3 and L4 to the slice below the perineum. The cropped volumes were then interpolated to be synCT and planning CT with an in-plane resolution of 1.25 × 1.25 mm^2^ and a slice thickness of 2.5 mm as required for RT planning. Finally, a deformable registration was performed between synCT and CT using the mutual information-based image registration in Velocity software (Velocity 3.2, Varian Medical Systems).

### Treatment planning

Both registered synCT and CT were imported into a commercial treatment planning system Eclipse version 13.7 (Varian Medical Systems, Palo Alto, CA). Organs at risk (OARs) including small bowel, bladder, and femoral head were contoured for each patient on overlapped synCT and CT view. Body contour generated automatically from synCT in Eclipse was used as a common body structure for both synCT and CT images. A radiation oncologist specialized in gastro-intestinal disease contoured a hypothetical clinical target volume (CTV) in rectal region, representing typical rectal tumor. A planning target volume (PTV) was then created by adding 1 cm margin uniformly to the CTV.

Commonly-used treatment techniques including both 3DCRT and VMAT were planned on synCT to treat the rectal target volume. The planning used a typical clinical protocol that prescribed 50.4 Gy to PTV with a fractional dose of 1.8 Gy in 28 fractions. The conformal plan was implemented by a 4-field box technique (4F) that delivered static radiation fields at cardinal gantry angles of 0°, 90°, 180° and 270°. To evaluate the effect of bone content inside beams, an oblique 4-field radiation (O4F) which had gantry angles of 45°, 135°, 225° and 315° was also planned, as is often performed for prosthetic patients and recommended by TG-63 [29]. The VMAT plan used two 360° arc fields and optimized using Eclipse progressive resolution optimization (PRO 13.7.1) algorithm to fulfill dose constraints in the protocol.

The three plans on each patient were copied to CT data set for dose calculation with the same MUs used in the synCT plans. The analytical anisotropic algorithm (AAA 13.7) was used to calculated dose on both synCT and CT with heterogeneity corrections [30]. We also calculated VMAT plans on both images using Acuros XB algorithm (Acuro 13.1) to evaluate the impact of calculation algorithm on synCT-to-CT dosimetric comparison, as Acuros XB was reported to be superior in dose calculation than the AAA in heterogeneous phantom and various sites [31–35]. All volumetric dose calculations were set with a grid resolution of 2.5 × 2.5 × 2.5 mm^3^.

### Dosimetric evaluation

To assess whether synCT and CT were equivalent for treatment planning, dose-volume histogram (DVH) and associated metrics were assessed for target volume and OARs in each plan. Based on our clinical practice, OAR doses were evaluated as: V40Gy and mean dose for bladder, V45Gy for small bowel, and V30Gy for femoral head, where V*x*Gy is the percentage volume of the OAR that receives dose ≥ *x* Gy. PTV dose was assessed by D100%, D95%, D2% and mean dose, where D*x*% was percentage dose (relative to prescription dose) in the PTV that covered *x*% volume. Medians, first (Q1) and third quantiles (Q3) of the metrics were calculated from the eleven patients’ data. Mann-Whitney non-parametric U-test was performed to evaluate the differences of these metrics between synCT and CT plan using a same treatment technique. A significant level *p = 0*.*01* was used for the multiple tests. Furthermore, the graphic technique assessing agreement between two measurements [36] was applied to test the equivalence of the metrics between synCT and CT. The limit of agreement [36] was defined as (Mean– 2STD, mean + 2STD) of the metric differences, where STD is standard deviation. A tolerance of 2% was selected as the criterion for acceptance.

Spatial dose distributions were compared by absolute dose differences between synCT and CT plans for dose grid points with a dose greater than 10% prescription dose. Mean and STD of the differences in a patient volume were calculated. Moreover, 3D gamma analysis of dose distributions between synCT and CT was performed at dose-difference and distance-to-agreement criteria of 1%/1mm and 2%/2mm with a 10% dose threshold. The analysis generated a gamma map where a gamma value < 1 indicated that the point had comparable dose. A passing rate was calculated from the gamma map as the percentage of dose points that met the criteria (i.e., gamma value < 1). All comparisons for dose distributions were performed on both AAA and Acuros XB calculated plans. Non-parametric U test with *p = 0*.*01* was performed to compare the means of the volumetric dose differences between dose calculation algorithms.

## Results

**Fig 1:**
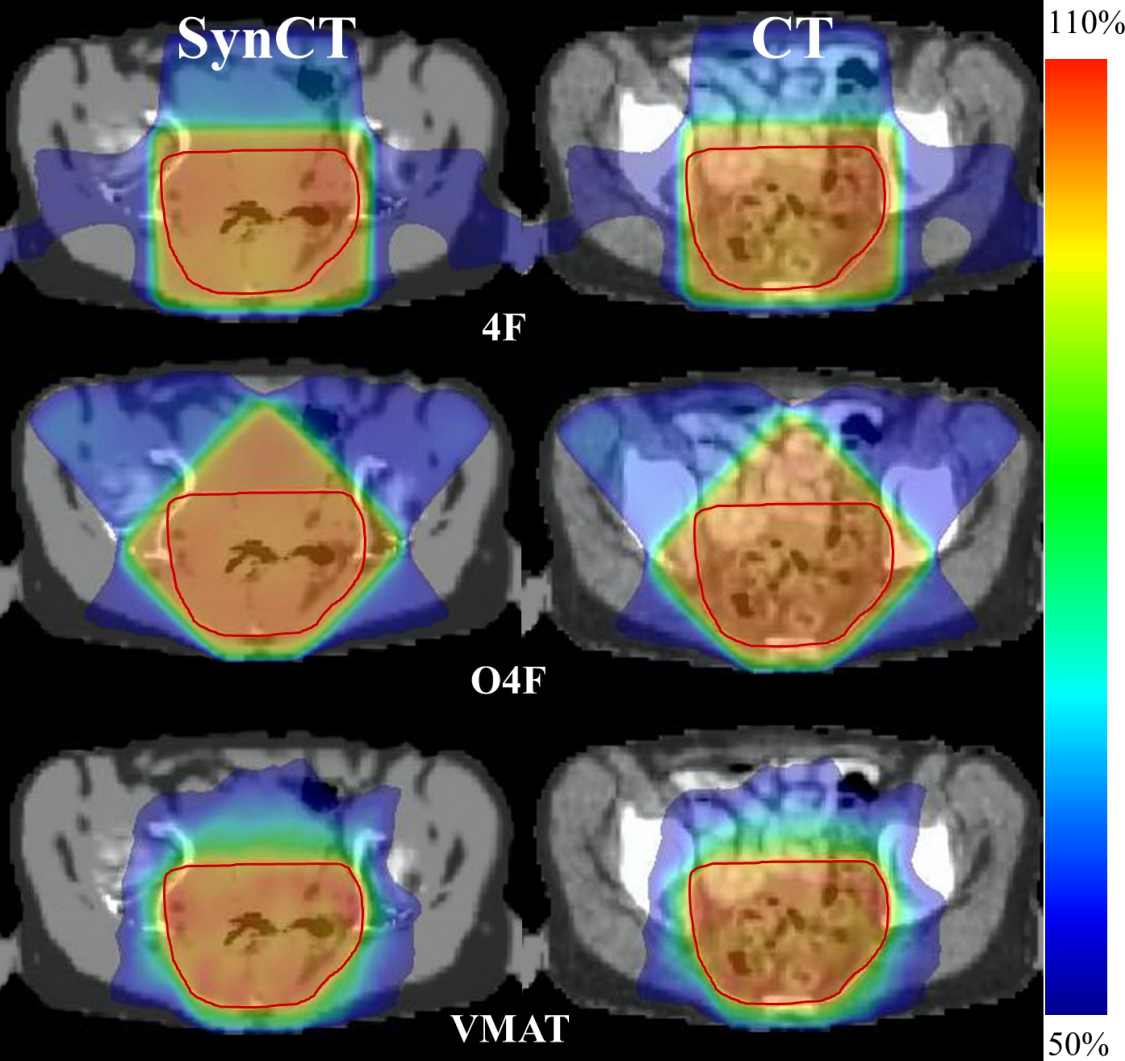
Doses of 4F (top), O4F (middle) and VMAT (bottom) plan overlapped on synCT (left column) and CT (right column). The doses are color mapped between 50% and 110% of prescription dose. PTV is contoured as red curve in color wash.

The volumes of the patient PTVs varied in a range of 759–1204 mm^3^ (mean ± std: 963 ± 148 mm^3^). Colorwash doses along with the PTV for a representative patient #5 is shown in Fig 1 for various techniques. All three plans accomplished the planning goal of 50.4Gy prescription dose covering 95% PTV. The VMAT plan showed smaller doses in the tissue surrounding PTV due to plan optimization using arc beams. While O4F showed less radiation in the femoral bone as expected than the 4F plan, O4F had more doses in the bladder and bowel regions.

**Fig 2:**
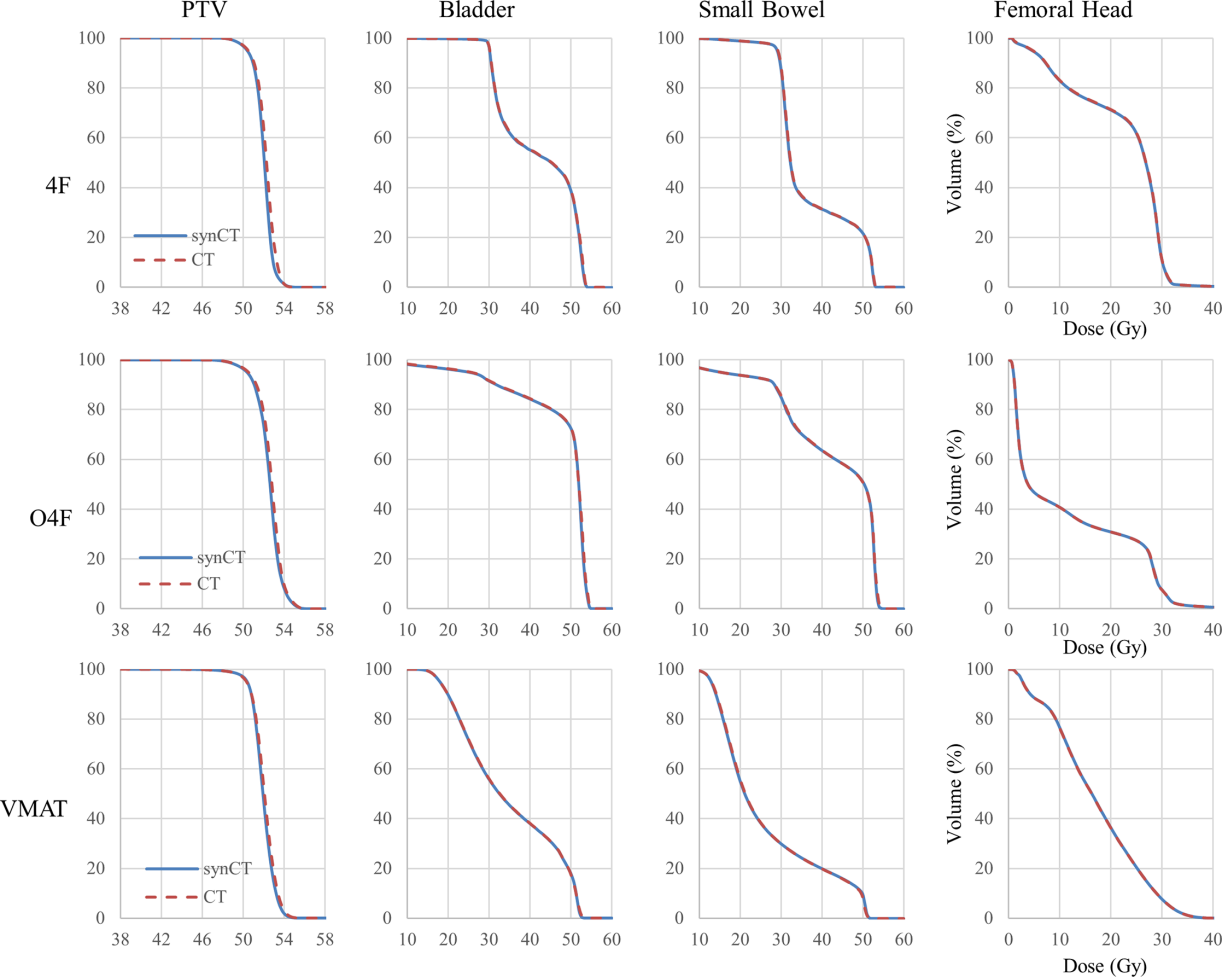
Comparison of average DVHs between synCT and CT plans. In each plot, horizontal axis is dose in Gy, and vertical axis is volume percentage for a DVH curve.

**Fig 3:**
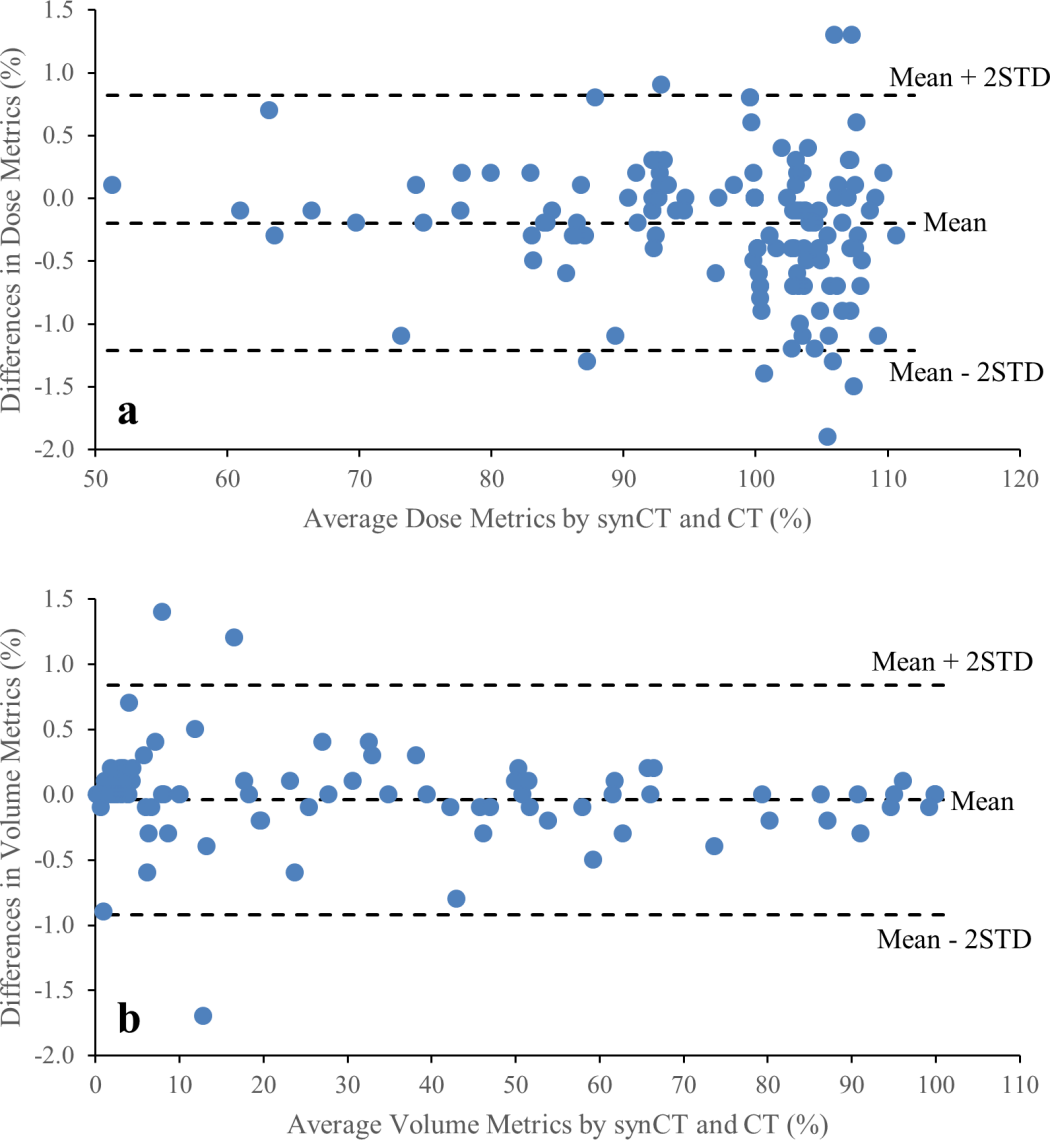
synCT-to-CT differences against metric average from synCT and CT. (a) was for dose metrics of PTV D100%, D95%, D2%, Dmean and Bladder Dmean, (b) was for volume metrics of Bladder V40Gy, Bowel V45Gy and Femoral Head V30Gy. The limit of agreement was calculated as (Mean-2STD, Mean+2STD) of the metric differences.

Average DVH curves for PTV and OARs from all the patients are shown in Fig 2. The average DVH curves were almost identical between synCT and CT plans. Dose metrics derived from the DVHs are listed in Table 1. Regardless of treatment technique, U tests suggested no significant difference in any PTV metric between synCT and CT plan. The OAR dose metrics present considerable variations between patients, but none showed significant differences between synCT and CT. Metric differences against means of the metrics from synCT and CT in all three treatment techniques were plotted in Fig 3 for statistical agreement assessment. The mean difference for the dose metrics (PTV D100%, D95%, D2%, Dmean and Bladder Dmean) was -0.2% with 95% interval -1.2% to 0.8% (Fig 3A). Fig 3B showed the limit of agreement for volume metrics (Bladder V40Gy, Bowel V45Gy and Femoral Head V30Gy) was (-0.9%, 0.8%), indicating the agreement between synCT and CT doses.

**Table 1:** Comparison of dose metrics for PTV and OARs. Medians, 1^st^ and 3^rd^ quantiles of the metrics from a plan are listed for each treatment technique, *p*-value is from Mann-Whitney U test of the dose metric between synCT and CT plans.

		4F			O4F			VMAT		
		synCT	CT	*p*-value	synCT	CT	*p*-value	synCT	CT	*p*-value
PTV	D100%	93.2	92.7	0.53	92.1	92.2	1.00	86.2	86.5	0.77
		(92.7, 93.4)	(92.7, 93.2)		(91.1, 92.5)	(91.1, 92.5)		(82.9, 87.5)	(83.3, 87.7)	
	D95%	100.0	100.0	0.45	100.0	100.0	0.11	100.0	100.0	1.00
		(100.0, 100.0)	(100.0, 100.2)		(100.0, 100.0)	(100.0, 100.6)		(100.0, 100.0)	(99.7, 100.5)	
	D2%	105.3	106.1	0.72	107.9	107.9	0.72	107.1	107.4	0.16
		(104.9, 107.1)	(105.1, 107.0)		(106.7, 108.9)	(106.8, 109.4)		(106.7, 107.3)	(107.0, 108.1)	
	Dmean	103.2	103.5	0.24	104.4	104.6	0.39	103.1	103.2	0.35
		(102.8, 103.4)	(103.1, 104.0)		(103.9, 104.6)	(103.8, 105.1)		(102.9, 103.3)	(103.0, 103.7)	
Bladder	V40Gy	59.0	59.5	0.95	94.6	94.7	1.00	42.2	42.3	0.95
		(35.4, 73.2)	(35.4, 73.3)		(87.4, 100.0)	(87.3, 100.0)		(25.6, 48.7)	(26.0, 48.7)	
	Dmean	85.4	86.0	0.90	100.9	101.2	0.95	69.7	69.9	0.87
		(75.4, 89.5)	(75.3, 89.5)		(97.6, 102.5)	(97.8, 102.5)		(63.0, 76.2)	(63.4, 76.4)	
Bowel	V45Gy	19.5	19.7	0.97	62.6	62.9	1.00	8.5	8.8	0.91
		(7.4, 45.2)	(7.4, 45.2)		(38.8, 73.0)	(38.7, 72.9)		(4.3, 27.0)	(4.1, 26.9)	
Femoral Head	V30Gy	8.7	7.3	1.00	3.9	3.7	0.90	5.9	6.5	1.00
		(2.2, 17.4)	(2.2, 16.5)		(2.2, 14.9)	(2.0, 15.0)		(3.4, 10.7)	(3.4, 10.9)	

Dmean: percentage mean dose relative to prescription dose

**Fig 4:**
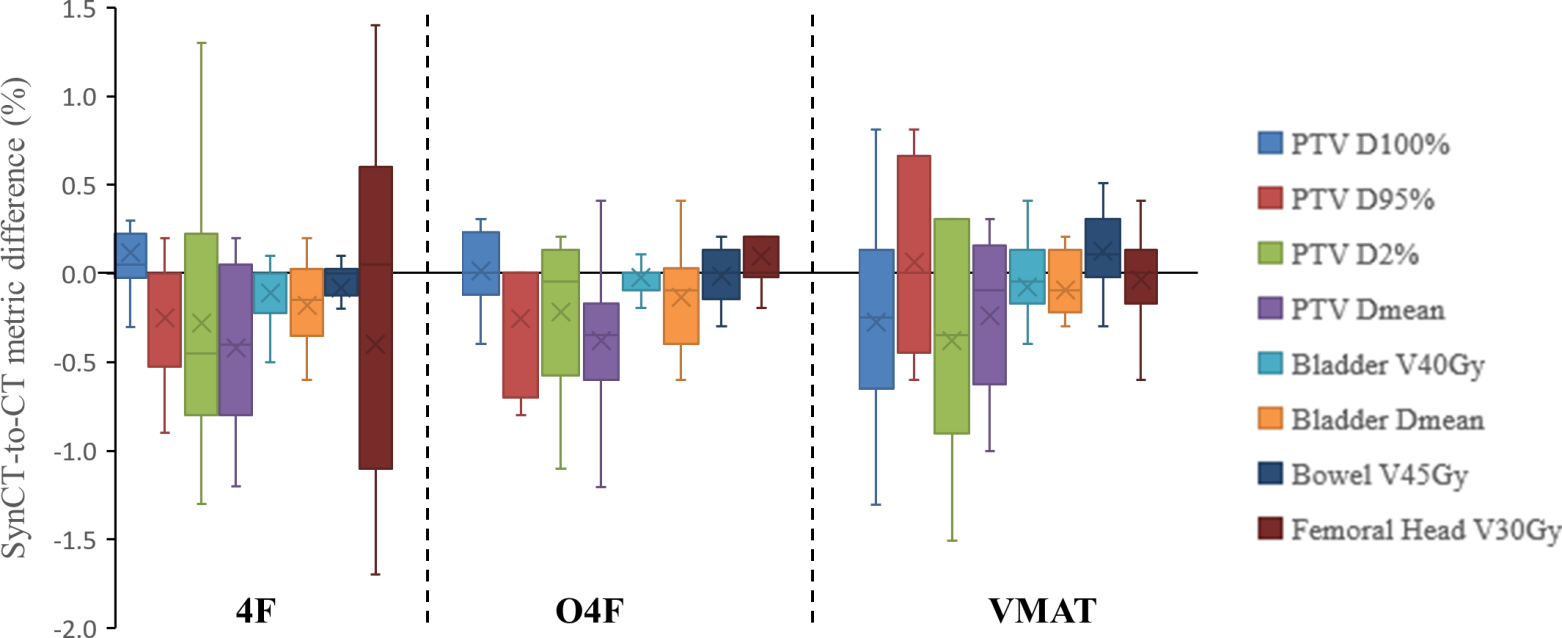
Box and whisker plot of differences in dose metric averages between synCT and CT plan for three treatment techniques. A box showed 25^th^ and 75^th^ percentiles, the whisker indicated maximal and minimal values, the line inside a box indicates median, and the cross is mean of the differences in dose metric averages in the eleven patients.

The synCT-to-CT differences in the dose metrics are plotted in Fig 4 for each plan technique. Medians of the absolute differences in all evaluated PTV metrics were 0.2% (Q1-Q3: 0.1–0.7%) for 4F, 0.2% (0.1–0.4%) for O4F and 0.3% (0.2–0.6%) for VMAT plan. Meanwhile, in all treatment techniques (total 30 plans), the differences in bladder V40Gy, bowel V45Gy and femoral head V30Gy were 0.1% (0–0.2%), 0.1% (0–0.3%), 0.2% (0–0.4%), respectively.

**Fig 5:**
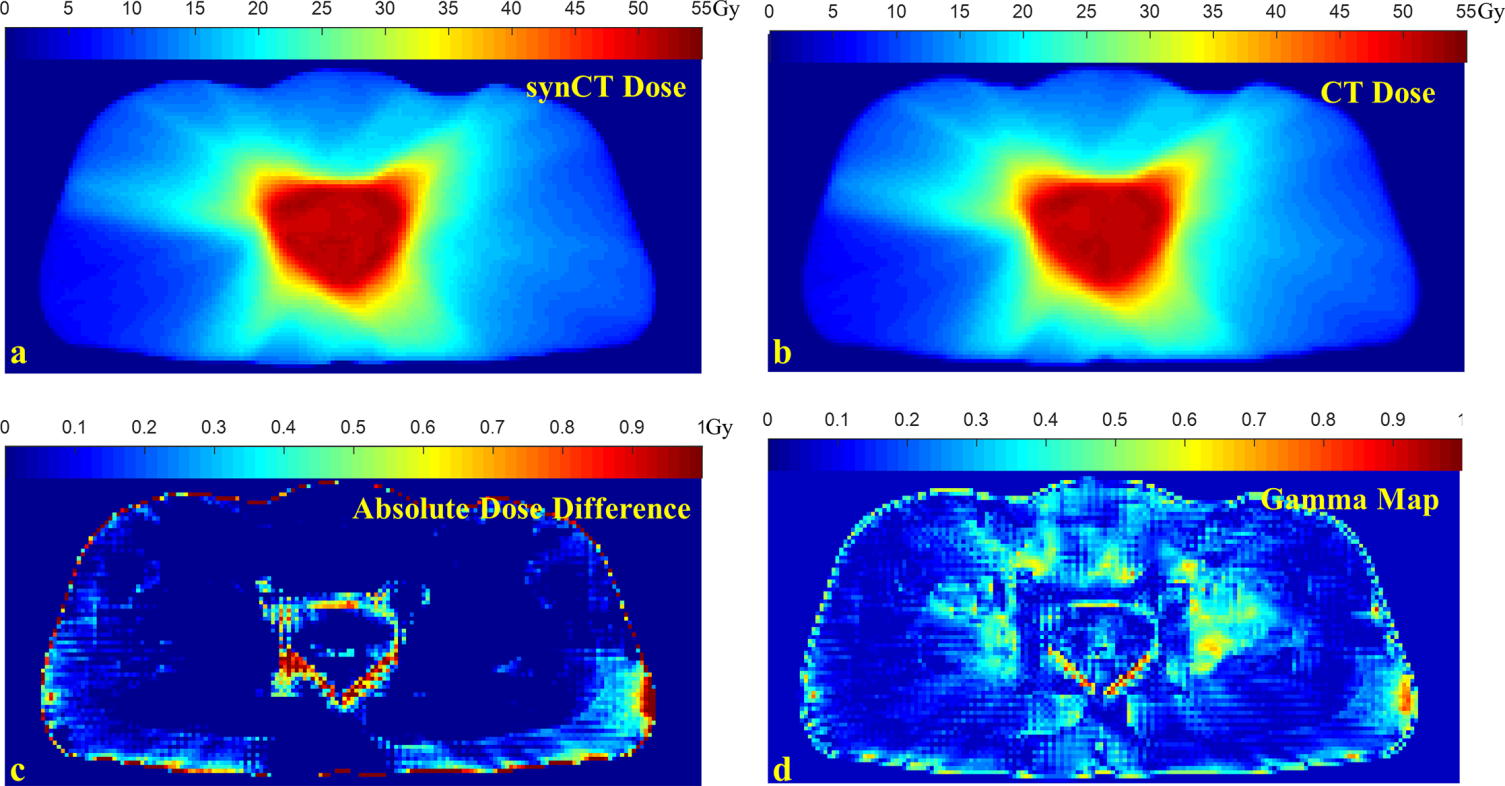
Comparison of VMAT plan dose distributions. a: dose calculated on synCT; b: dose calculated on CT; c: absolute dose difference for the dose slice; d: gamma map of 2%/2mm gamma analysis for the synCT and CT dose slice.

A dosimetric analysis for a VMAT plan is shown in Fig 5. Fig 5C shows absolute dose difference of the slice between synCT and CT plan with a scale from 0 to 1 Gy, on which the dose differences is less than 0.2 Gy (i.e., <0.4% prescription dose) in most part of the body. The points with dose differences greater than 1 Gy were present at the edges of the body and PTV. The gamma map from 2%/2mm gamma analysis of the slice is shown in Fig 5D with a gamma value scale from 0 to 1, indicating almost all the points had equivalent doses between synCT and CT plan.

**Fig 6:**
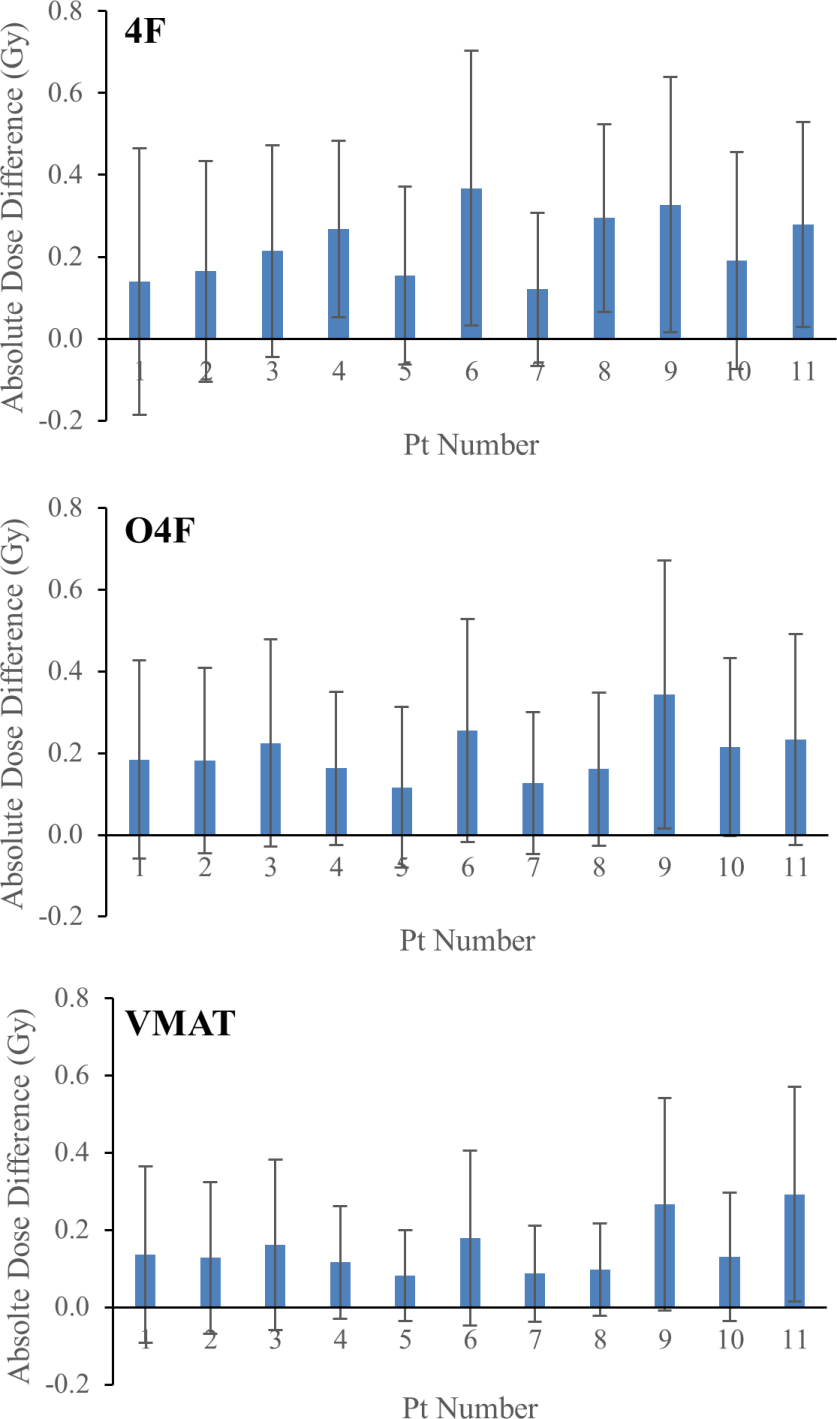
Average of volumetric absolute dose differences between synCT and CT plan for each patient.

Fig 6 compares volumetric averages of absolute synCT-to-CT dose differences for each patient. Medians of the dose difference averages were 0.21 Gy (0.16–0.29 Gy), 0.18Gy (0.16–0.23Gy) and 0.13Gy (0.11–0.17Gy) for 4F, O4F and VMAT, respectively. 1%/1mm gamma analysis also showed that the passing rate from VMAT is ~2% higher than those from 3DCRTs (Table 2). Regardless of planning techniques, the median passing rates between synCT and CT plans (Table 2) were higher than 96% and 99% for 1%/1mm and 2%/2mm criteria, respectively.

**Table 2:** Passing rates of gamma analysis for synCT and CT-calculated dose distributions for each patient.

	4F		OBL 4F		VMAT	
Patient ID	1%/1mm	2%/2mm	1%/1mm	2%/2mm	1%/1mm	2%/2mm
1	99.4	100.0	97.5	99.9	99.0	99.9
2	97.9	99.8	99.1	100.0	98.9	100.0
3	97.0	99.7	96.7	99.9	98.0	99.9
4	93.1	99.7	96.6	99.7	98.8	99.9
5	99.0	100.0	99.7	100.0	100.0	100.0
6	87.9	98.5	94.6	99.7	97.4	99.7
7	99.6	99.9	100.0	100.0	100.0	100.0
8	90.6	99.8	98.4	100.0	99.9	100.0
9	88.1	99.0	85.4	97.2	93.7	99.6
10	98.1	100.0	96.5	100.0	99.0	100.0
11	92.5	99.6	95.5	98.7	91.9	99.4
median (range)	97.0 (87.9–99.6)	99.8 (98.5–100.0)	96.7 (85.4–100.0)	99.9 (97.2–100)	98.9 (91.9–100.0)	99.9 (99.4–100.0)

We re-calculated the VMAT plans on synCT and CT using the Acuros XB algorithm that may improve patient dose estimation [34]. Table 3 summarizes dose differences and gamma analysis of Acuros XB-calculated VMAT plans. Using Auros XB, 2%/2mm gamma analysis of VMAT plans showed 99% pass rating between synCT and CT, along with a median of the dose difference averages 0.20 Gy (~0.4%, range: 0.13–0.64 Gy), suggesting the clinical agreement between synCT and CT plans, irrespective of dose calculation algorithm.

**Table 3:** Comparison of dose distributions between VMAT plans using AAA and Acuros XB calculations on synCT and CT.

Comparison between plans		Median (Q1, Q3) of dose difference average (Gy)	Median (range) of gamma passing rates (%)	
			1%/1mm	2%/2mm
synCT.vs. CT	AAA	0.13 (0.11, 0.17)	98.9 (91.9–100.0)	99.9 (99.4–100.0)
	Acuros XB	0.20 (0.16, 0.23)	96.3 (89.9–98.4)	99.2 (97.7–99.9)
AAA.vs. Acuros XB	CT	0.38 (0.35, 0.40)	93.4 (90.6–95.7)	99.7 (99.7–99.9)
	SynCT	0.38 (0.35, 0.41)	93.6 (87.8–94.7)	99.8 (99.6–99.9)

Table 3 also compares doses between AAA and Acuros XB on a same image. On either synCT or CT, 2%/2mm gamma analysis on doses between two algorithms had >99% passing rates, indicating their equivalence for clinical dose calculation. 1%/1mm gamma analysis showed ~93% passing rates between AAA and Acuros XB, which were slightly smaller than those (> 95%) from dose comparisons between synCT and CT. The means of AAA-to-Acuros XB dose difference averages were ~0.4Gy (~0.8%). Mann-Whitney U tests on the dose difference averages further demonstrated that synCT-to-CT dose differences using either algorithm were significantly (*p* < 0.001) smaller than AAA-to-Acuros XB dose differences on either image.

## Discussion

This study showed that synCT generated from Dixon-MR using an automated model-based method provided accurate and equivalent dose calculation as conventional CT for treatment planning of rectal cancer. The study performed treatment planning on synCT as a MR-only workflow, and then recalculated the plans on CT for evaluation. Dosimetric agreement between synCT and CT was demonstrated by the similarities in spatial dose distributions as well as PTV and OARs dose metrics. The results suggest that MR-generated synCT can support a MR-only workflow for radiotherapy of rectal cancer by providing electron density map like conventional CT for accurate dose calculation.

The MR generated synCT has been evaluated for treatment planning of VMAT for brain [18] and liver tumors [19], IMRT for head and neck cancer [37], prostate tumor [20] and lung cancer [25]. Recently, a commercial synCT software has been evaluated for use in prostate radiotherapy [38]. In this study, we demonstrated that MR generated synCT was sufficient for planning of both 3DCRTand VMAT of rectal cancer. The clinical equivalence between synCT and CT plan was assessed by dose metrics that physicians use to evaluate a plan for rectal cancer treatment. Voxelwise dose differences further indicated the close matching of dose distributions from the two modalities. Fig 5 shows that the voxels having a relatively high dose difference mostly located at the high dose-gradient edge regions where the dose differences were sensitive to possible misalignment between synCT and CT. The spatial error was accounted for by a distance-to-agreement criterion in gamma analysis which is widely used for comparison of dose distributions [39]. The high passing rates of gamma analysis in Table 2 indicated that synCT calculated doses at these voxels, although exhibiting dose discrepancies, agreed with CT calculated dose distribution with clinically-used dose-difference/distance-to-agreement criteria. Acuros XB was found to provide more accurate dose calculation than AAA near air-tissue interface [34, 35, 40]. We thus evaluated AAA and Acuros XB dose calculations in this study as substantial air-tissue interface presents in rectum. Gamma analysis in Table 3 showed that synCT and CT calculated doses well agreed with each other for both Acuros XB and AAA. Acuros XB is now gradually gaining use in clinic for dose calculation. Table 3 showed that the dose discrepancy between AAA and Acuros XB calculation was greater than the difference between synCT and CT dose using either algorithm. These further suggested the clinical equivalence of synCT and CT for radiation dose calculation.

A hybrid method [24] was used in this study to generate synCT from MR images. The method avoids the difficulty of differentiating between bone and air in the classification-based method and the uncertainty in registration of soft tissue in the atlas-based method. The method still utilizes registration between patient MR images and atlas for bone delineation. The registration has been refined in the method by first a landmark matching and then an intensity-based deformable registration since the atlas is only for bone [24]. Nevertheless, the bone could be prone to registration uncertainty and can lead to dose calculation error when irradiating through bone. A VMAT plan delivered IMRT fields during a continuous gantry rotation. Compared with 4F conformal RT, a much smaller portion of the VMAT MU irradiated through pelvic bone. Therefore, as indicated in the Table 2, VMAT showed a slightly higher passing rate than the 3D plans for 1%/1mm gamma analysis. Despite the difference in 1%/1mm gamma analysis, our results showed that the dose differences between synCT and CT were small and clinically insignificant regardless of the treatment techniques. Recently, a synCT generation method has been reported to identify pelvic bone by using a pelvic bone shape model [21]. Similarly, bone identification from the shape model was prone to matching error, but clinically, the generated synCT was promising for treatment planning.

Image registration between synCT and CT was performed in this study to alleviate potential tissue displacement from PET/MRI to PET/CT acquisition. The uncertainty in the registration could lead to minor misalignment near body contour and organ edges between synCT and CT images, which may amplify the dose differences at high dose-gradient area (Fig 5C). Additionally, we observed differences in the level of rectal and bladder filling between MR and CT images. These may add extra errors in current results of dosimetric comparison between synCT and CT plans. Practically, use of the synCT in a MR-only workflow will avoid the CT scan, possible artifacts in CT images, and the uncertainty of MR/CT registration.

The synCT was generated by assigning a HU number to a voxel based on its MR-derived tissue type. It ignored the variation of HU numbers within a tissue type and between patients. Various studies have shown that radiation dose deposition is relatively insensitive to tissue HU number [16, 20, 41]. It was reported that HU differences smaller than 34 HU would not affect dosimetric result [16, 42]. In addition, synCT should be geometrically accurate to represent patient anatomy for dose calculation. However, MR image is known to have geometrical distortion. The distortion depends on numerous sources including the MR scanner, field strength, pulse sequences and image FOV, and the magnitude could be substantially greater than desired spatial accuracy for a radiation simulator [43]. Geometrical distortion for the rectum near scanner isocenter may be insignificant, but could be problematic at body surface for an oversize patient. Fortunately, phantoms and distortion correction methods considering specific requirements for radiotherapy are increasingly being provided by vendors [43, 44]. Various studies have shown that the distortions can be reduced to the acceptable level for radiation treatment in many sites [14, 45]. Undoubtedly, comprehensive quality assurance of MR geometrical distortion is an essential prerequisite for clinical application of MR-generated synthetic CT.

As the emergence of MR-Linac technique and development of MR-guided radiotherapy, a MR-only treatment planning will spare the extra effort of CT simulation and simplify treatment workflow with cost effectiveness. Implementation of MR-only treatment planning using synCT is being evaluated clinically in several institutions [12, 19, 45, 46]. This study comprehensively evaluated the synthetic CT for treatment planning in eleven patients. It is limited by the relatively small number of patients, but nevertheless supports further development of a MR-only treatment planning for rectal cancer.

## Conclusion

Treatment planning on MR generated synCT agreed very well with the dose calculated on CT for 3DCRT and VMAT planning for rectal cancer treatment. Mean dosimetric difference between synCT and CT was less than 0.4% for any treatment technique. The study demonstrated the potential of MR-only treatment planning for colorectal cancer thus streamlining the patient care.
